# Myocardial metabolic remodeling in human end-stage ischemic and non-ischemic cardiomyopathy

**DOI:** 10.1016/j.yjmcc.2026.02.008

**Published:** 2026-02-26

**Authors:** Hongyi Zhou, Courtney Jayde Christopher, Katarina Jones, Michelle Mendiola Pla, Ryan T. Gross, Gabriel Esmailian, Shawn Robert Campagna, Dawn E. Bowles, Weiqin Chen

**Affiliations:** a Department of Physiology, Medical College of Georgia, Augusta, GA, USA; b Department of Chemistry, The University of Tennessee, Knoxville, TN, USA; c The Biological Mass Spectrometry Core, The University of Tennessee, Knoxville, TN, USA; d Department of Surgery, Duke University Medical Center, Durham, NC, USA

**Keywords:** Heart failure, Metabolomics, Ischemic cardiomyopathy, Nonischemic cardiomyopathy

## Abstract

**Background:**

Heart failure (HF) is linked to disturbances in heart metabolism. Metabolomics shows promise in identifying cardiac-specific metabolic changes across different types of heart disease. However, direct comparison of metabolomic changes in myocardial tissues from humans with end-stage ischemic (ICM) and nonischemic (NICM) heart failure is scarce.

**Methods:**

Left ventricles were collected from patients with end-stage ICM (*n* = 14) and NICM (*n* = 15), along with nonfailing donors (*n* = 11). Untargeted metabolomics assessed organic acids, amino acids, purines, and pyrimidines. Data were analyzed using partial least squares-discriminant analysis (PLS-DA), heat maps, Kruskal-Wallis with Dunn test, and ANOVA. RT-PCR and Western blotting were used to examine the expression of metabolic genes.

**Results:**

Myocardial metabolites could distinguish diseased from nonfailing hearts; however, ICM and NICM samples often overlapped, despite ICM patients having higher rates of diabetes and hyperlipidemia. Glycolytic and TCA metabolites showed no differences within the groups. Notably, myocardial UDP-N-Acetyl-glucosamine and O-GlcNAcylation levels were consistently lower, despite increased expression of hexosamine pathway genes in both disease groups. Several amino acids decreased, yet branched-chain amino acids remained stable in ICM and NICM hearts. Both groups showed hyperhomocysteinemia and increased urea cycle intermediates. Glutathione (GSH) and glutathione disulfide (GSSG) levels were depleted regardless of glutathione synthesis gene expression. Adenylated purines and pyrimidines were reduced, with increased purine degradation and a notable upregulation of NC5E, an extracellular nucleotidase, in both disease groups. While ATP and NAD+ levels stayed relatively stable, NADH, FAD+, and NADP+ levels decreased in diseased hearts. Catalase was upregulated in both disease groups despite elevated markers of oxidative stress.

**Conclusion:**

Human end-stage heart failure is characterized by altered glucose and amino acid metabolism, heightened oxidative stress, increased purine breakdown, and reduced pyrimidine levels, with no differences observed between ischemic and nonischemic cardiomyopathy. These findings enhance our understanding of metabolic alterations in failing human hearts.

## 1. Introduction

Heart failure (HF) is a global health issue with significant mortality and morbidity [1]. Despite advances in early diagnosis and new treatment options, HF remains one of the most difficult diseases to manage. Patients with HF are often classified by left ventricular ejection fraction (LVEF). There are three subtypes of HF: heart failure with preserved ejection fraction (HFpEF ≥50%), heart failure with mildly reduced ejection fraction (HFmrEF, 41–49%), and heart failure with reduced ejection fraction (HFrEF, EF ≤ 40%) [2]. HF is also broadly categorized into ischemic and non-ischemic cardiomyopathy based on its underlying cause. Ischemic cardiomyopathy (ICM) results from coronary artery disease and myocardial infarction, which cause decreased blood flow and oxygen supply to the heart muscle, leading to systolic LV dysfunction [3]. Non-ischemic cardiomyopathy (NICM), on the other hand, has various causes, including genetic factors, viral infections, toxic exposures, and idiopathic origins [4]. Patients with ICM tend to have worse outcomes than those with NICM [5]. However, current therapeutic strategies are mostly based on LVEF and do not account for the underlying HF etiology. Our understanding of metabolic reprogramming in different HF subtypes remains incomplete, underscoring the importance of elucidating disease mechanisms in patients’ myocardium to improve etiology-based HF management.

Disruptions in cardiac energy metabolism are closely associated with various forms of HF, as the heart is the body’s most energy-demanding organ [6]. Metabolomics is increasingly utilized to discover new disease pathways. Plasma metabolomics has identified significant metabolic disturbances associated with HF or increased mortality risk, supporting the idea that circulating metabolites can indicate underlying disease states [7–10]. Circulating metabolites from various pathways, including amino acids, nucleotides, lipids, and acylcarnitines, have been linked to HF risk [7–10]. Recent metabolomic profiling of myocardial tissue from patients with end-stage nonischemic dilated cardiomyopathy [11] and HFpEF [12] revealed key metabolic changes specific to HF subtypes. However, more myocardial metabolomic studies are necessary to compare changes across different HFrEF subtypes and to identify disease-specific metabolomic alterations for prognosis and their correlation with plasma metabolomics.

Our study directly compared metabolomic changes in ventricular tissues from end-stage ICM and NICM with nonfailing hearts to better understand myocardial metabolic profiles in HF caused by different etiologies. Although we did not find clear differences in the cardiac metabolome between ICM and NICM, we confirmed many previous findings and identified new metabolomic abnormalities in human end-stage HFrEF hearts. Analyzing these metabolic shifts can deepen our understanding of metabolic reprogramming in HF and potentially improve heart failure treatments and patient outcomes.

## 2. Methods

### 2.1. Human myocardial tissue procurement

Human tissue samples were obtained from subjects who consented and were collected by the Duke Human Heart Repository, which was approved by the Duke University Hospital System Institutional Review Board (IRB) (Pro00005621). A separate approval of “Exemption from IRB Review” was obtained from Augusta University IRB (2012–0460). De-identified samples were obtained, and data were presented without PHI. The following data were collected and presented per Duke’s IRB protocol: average age of the subject group at the time of tissue collection, gender, medical history (excluding rare diseases but including common concomitant medications), and common comorbidities. All patients in the failing heart group were diagnosed based on clinical criteria for end-stage heart failure. Detailed demographic and clinical characteristics of patients were presented in Table 1. Some of the explanted hearts had undergone LVAD implantation before transplantation. For controls, nonfailing heart samples from nonfailing donors that were ultimately not used for transplantation were obtained. Hearts were arrested in vivo using cardioplegic arrest and procured in standard fashion. Tissues were dissected and frozen in liquid nitrogen within 1–2 h.

### 2.2. Untargeted quantitative metabolomics

Metabolites from all samples were extracted and analyzed at the Biological Mass Spectrometry Core (BMSC) at the University of Tennessee, Knoxville (RRID: SCR_021368). Detailed descriptions of data processing and analysis were provided in the Supplemental Materials.

### 2.3. Total RNA isolation and quantitative RT-qPCR

Total RNA was extracted using TRIzol reagent (Thermo Fisher Scientific, Cat# 15–596–018) following the manufacturer’s protocol. For reverse transcription, MLV-V reverse transcriptase and random primers (Thermo Fisher) were used. RT-qPCR was conducted on the AriaMX system (Agilent) with SybrGreen dye. Gene expression levels were normalized to *RPLP0*. Results were presented as fold changes relative to nonfailing controls. Supplemental Table S1 provided the primer sequences used for RT-qPCR analysis.

### 2.4. Western blotting

Proteins were extracted, and standard Western blotting was performed. Detailed methods and antibody information are available in the Supplemental Materials.

### 2.5. Statistical analysis

Data were presented as mean values and SEM. All statistical analyses were performed using GraphPad Prism Ver. 10. The Kolmogorov-Smirnov test was used to assess normality. If the test was passed, a one-way ANOVA with Tukey’s multiple-comparison test was performed. Otherwise, the Kruskal-Wallis test with Dunn’s post hoc test was used.

The authors declare that all supporting data are available within the article and in the Supplemental Materials.

## 3. Results

### 3.1. Untargeted metabolomics reveal changes in metabolites in human ICM and NICM hearts

To characterize the metabolomic profiles of end-stage failing hearts with different causes, we performed untargeted metabolomic analysis of myocardial samples from 40 male patients: 11 nonfailing (NF) heart explants and 29 end-stage failing hearts from patients with ICM (*n* = 14) and NICM (*n* = 15). Among nonfailing controls, 72.7% reported a history of smoking, while 45.5% had hypertension without other comorbidities. Heart failure patients were slightly older but had a similar median BMI of 30 and smoking history to nonfailing controls. ICM patients showed higher rates of type 2 diabetes, hyperlipidemia, and chronic kidney disease than NICM patients. Atrial fibrillation was observed in 28% of ICM patients and about one-third of NICM patients (Table 1). More ICM patients were treated with insulin, whereas more NICM patients were treated with a β-blocker (Table 1).

**Fig. 1.**
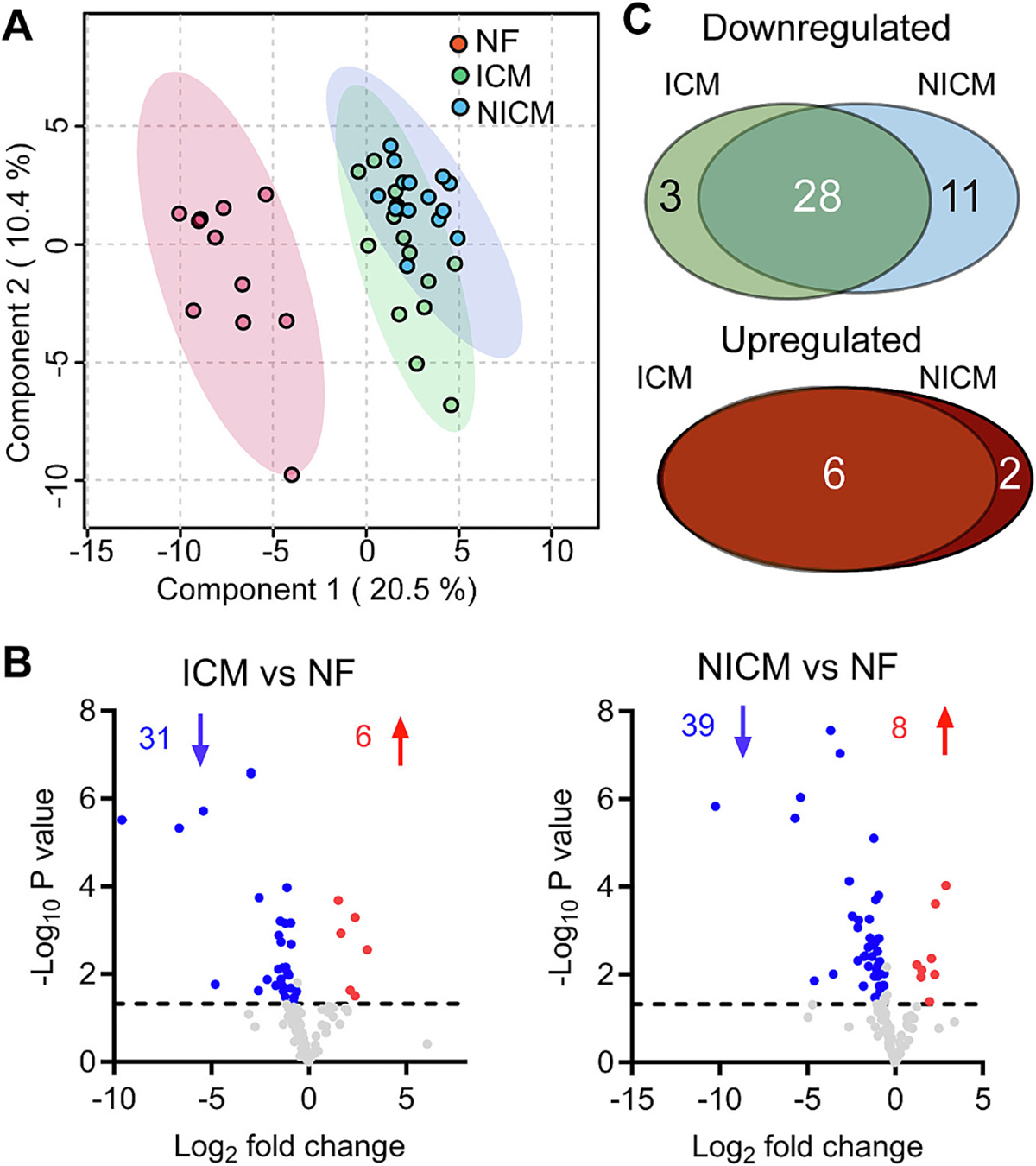
Cardiac metabolite changes in human ICM and NICM. A) Partial least squares discriminant analysis (PLS-DA) using MetaboAnalyst 5.0. shows the considerable separation of 11 nonfailing controls from 14 ICM and 15 NICM based on the untargeted quantitative metabolomics of myocardial tissues. B) Volcano plot of differential metabolites between ICM and nonfailing hearts, as well as NICM versus nonfailing hearts. C) Venn diagram showing overlapping downregulated and upregulated metabolites shared in both ICM and NICM groups. Differential metabolites were identified by an unpaired *t*-test with *P* < 0.05 and 0.59 ≤ Log_2_ fold changes (FC) ≤ −0.59 (i.e., 1.5 ≤ FC ≤ −1.5) when compared to nonfailing controls, respectively. NF; nonfailing, ICM: ischemic cardiomyopathy, NICM: nonischemic cardiomyopathy.

We identified 124 metabolites in the ventricles of all samples (Supplemental Excel). PLS-DA effectively separated nonfailing controls from patients with ICM or NICM but did not distinguish between ICM and NICM (Fig. 1A). When examining differential metabolites relative to nonfailing samples, ICM showed 31 downregulated and 6 upregulated metabolites, while NICM had 39 downregulated and 8 upregulated (Fig. 1B). 28 metabolites were commonly downregulated in both conditions, and all 6 metabolites upregulated in ICM myocardium were also elevated in NICM tissues. Additionally, two metabolites were uniquely increased in NICM groups (Fig. 1C). Despite these differences, direct comparisons between ICM and NICM showed no significance among them. These results suggest that the metabolic profiles of ICM- and NICM-failing hearts showed significant similarities in the final stages of heart failure.

### 3.2. Defective carbohydrate metabolism and maintained tricarboxylic acid metabolites in ICM and NICM hearts

**Fig. 2.**
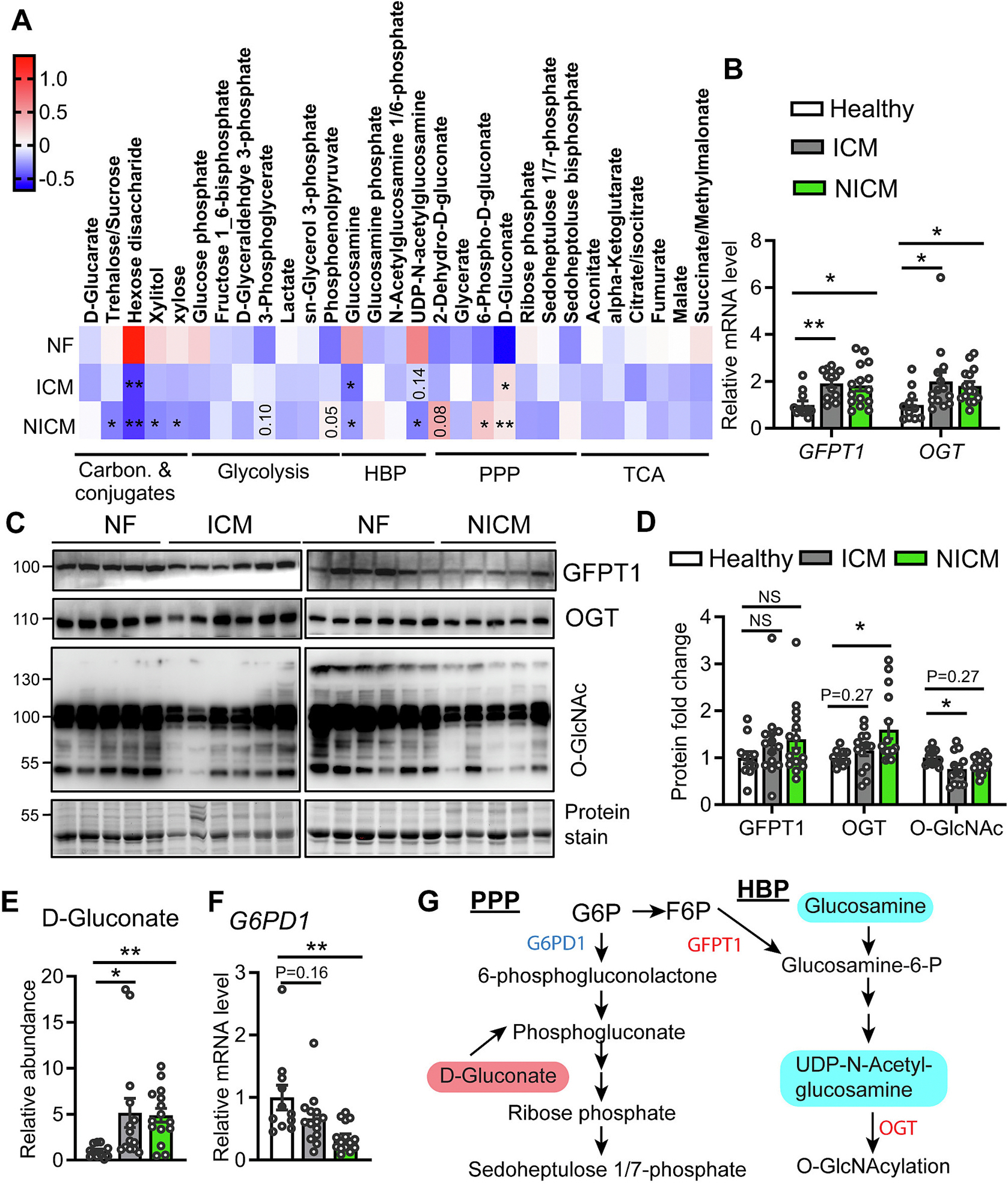
ICM and NICM hearts exhibit dysregulated metabolites related to carbohydrate metabolism and the TCA cycle. A) Heatmap illustrating metabolites categorized into carbohydrates and carbohydrate conjugates, glycolysis, hexosamine biosynthetic pathway (HBP), pentose phosphate pathway (PPP), and tricarboxylic acid cycle (TCA). Unpaired *t*-tests. B) RT-PCR analysis of *GFPT1* and *OGT* genes. Kruskal-Wallis test with post hoc Dunn test for multiple comparisons. C–D) Western blot and quantification of GFPT1, OGT and *O*-GlcNAcylation levels. Data was normalized to quantifications from stain-free bands. One-way ANOVA with Turkey’s post hoc test. E) Relative D-gluconate levels. Kruskal-Wallis test with post hoc Dunn test for multiple comparisons. F) RT-PCR analysis of G6PD1 gene expression. One-way ANOVA with Turkey’s post hoc test. *: P < 0.05; **: *P* < 0.005. G) Schematic diagrams of metabolites involved in HBP and PPP pathways. Upregulated metabolites and genes were highlighted in red; downregulated metabolites and genes were highlighted in blue.

The heart becomes unable to utilize glucose in end-stage HF [13]. In our human myocardial cohorts, we found NICM, but not ICM, myocardia showed a greater reduction in the accumulation of trehalose/sucrose and carbohydrate conjugates such as xylitol and xylose (Fig. 2A). Hexose disaccharides were almost undetectable in both ICM and NICM hearts (Fig. 2A). Among the intermediate metabolites in glycolysis and gluconeogenesis, only phosphoenolpyruvate (PEP) and 3-phosphoglycerate (3PG) showed a trend of being higher, especially in NICM, but no other metabolites, including lactate, displayed any significant changes (Fig. 2A). We also identified no differences in six metabolites of the tricarboxylic acid (TCA) cycle, other than a tendency of lower fumarate, malate and succinate in both ICM and NICM hearts (Fig. 2A).

Glucosamine bypasses glutamine-fructose-6-phosphate transaminase 1 (GFPT1) to enter the hexosamine biosynthetic pathway (HBP) and is converted to UDP-*N*-acetyl-glucosamine (UDP-GlcNAc) for O-GlcNAcylation (Fig. 2G). Glucosamine and UDP-GlcNAc were significantly lower in failing ICM and NICM hearts (Fig. 2A). The mRNA expressions of *GFPT1* and *O*-linked *N*-acetylglucosamine transferase (*OGT*), the key genes involved in *O*-GlcNAcylation, were significantly higher in both ICM and NICM ventricles (Fig. 2B). However, GFPT1 protein expression did not differ among the three groups, and OGT expression was slightly higher in the NICM hearts compared with nonfailing hearts (Figs. 2C–2D). Consistent with the reduced UDP-GlcNAc, the levels of *O*-GlcNAcylation were decreased in ICM hearts and showed a trend toward downregulation in NICM hearts compared to nonfailing controls (Figs. 2C–D).

Conversely, we observed increased levels of diet-derived d-gluconate in both ICM and NICM hearts (Figs. 2A & 2E), since human hearts do not directly oxidize glucose to gluconate. [14] Interestingly, two other intermediates (6-phospho-d-gluconate and 2-dehydro-d-gluconate) were specifically elevated or trended higher in NICM hearts, not in ICM hearts (Fig. 2A). However, the mRNA expression of glucose 6-phosphate dehydrogenase 1 (*G6PD1*), the rate-limiting enzyme of the oxidative pentose phosphate pathway (PPP) pathway, trended lower in ICM and was even significantly reduced in NICM compared to nonfailing controls (Fig. 2F). Overall, these findings indicate that end-stage HFrEF hearts maintained glycolytic and TCA cycle metabolites but showed reduced myocardial UDP-GlcNAc and O-GlcNAcylation, with minimal changes in PPP pathways (Fig. 2G).

### 3.3. Reduced amino acids, elevated homocysteine, and depletion of the glutathione pool in ICM and NICM hearts

**Fig. 3.**
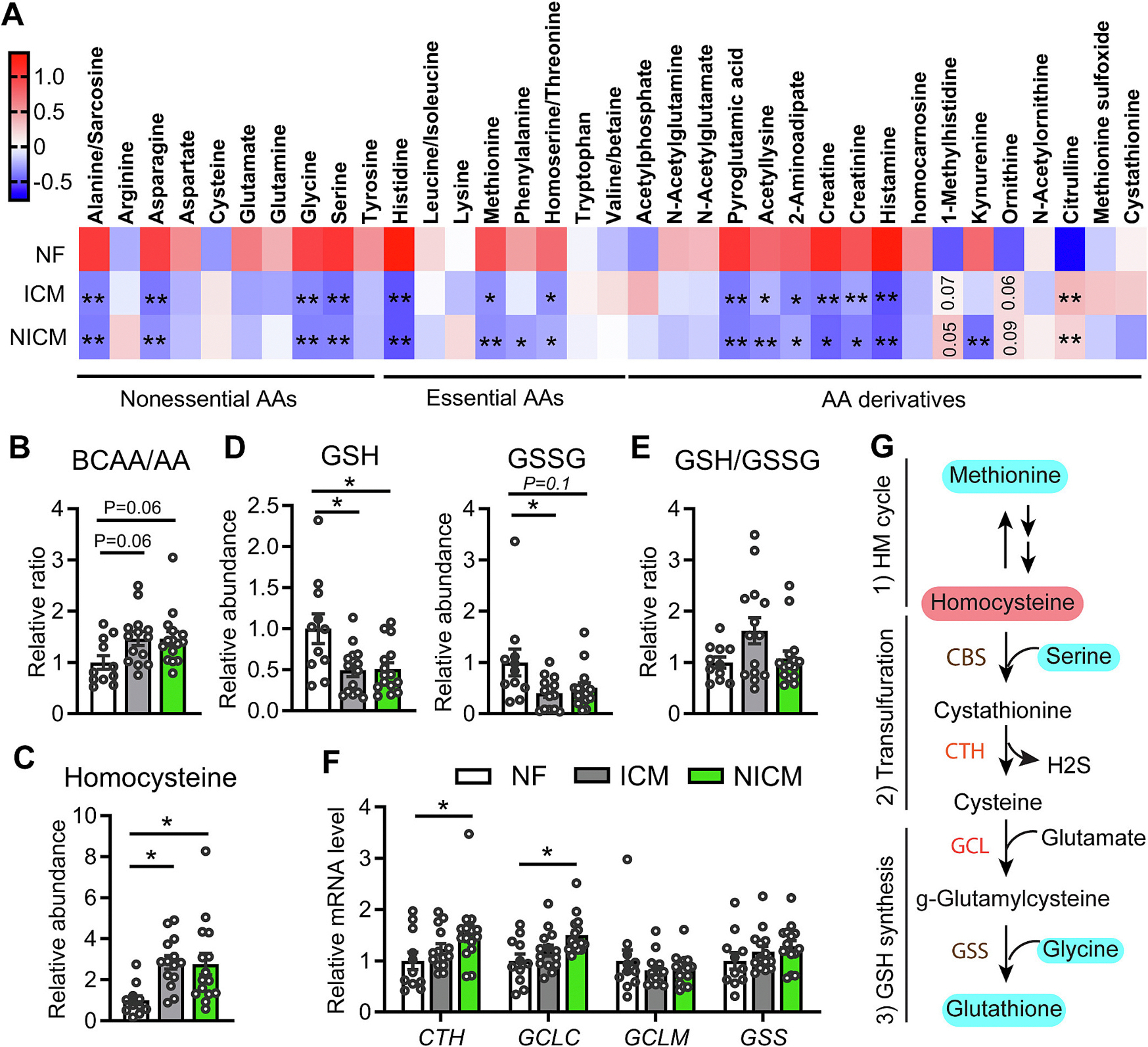
Myocardia from ICM and NICM exhibit reduced amino acids, elevated homocysteine, and depletion of the glutathione pool. A) Heatmap illustrating metabolites categorized into nonessential and essential amino acids and amino acid derivatives. Unpaired *t*-tests. B) Relative ratio of BCAA to total AAs. The ratio was calculated by the sum of BCAAs to the sum of the identified 19 AAs. One-way ANOVA with Turkey’s post hoc test. C) Relative abundance of homocysteine. One-way ANOVA with Turkey’s post hoc test. D) Relative abundances of GSH and GSSG. Kruskal-Wallis test with post hoc Dunn test for multiple comparisons. E) GSH/GSSG ratio. Kruskal-Wallis test with post hoc Dunn test for multiple comparisons. F) RT-PCR analysis of genes involved in glutathione synthesis. One-way ANOVA with Turkey’s post hoc. 11 nonfailing, 14 ICM, and 15 NICM hearts were analyzed. *: P < 0.05; **: *P* < 0.005. G) Schematic diagram of identified metabolites involved in the methionine/homocysteine (HM) cycle, transulfuration, and glutathione synthesis pathways. Upregulated metabolites and genes are highlighted in red; downregulated metabolites are highlighted in blue.

HF is well associated with disruptions in amino acid metabolism [15]. We identified 19 out of 20 amino acids in the myocardium, excluding proline (Fig. 3A). Several AAs, including nonessential AAs like alanine, asparagine, glycine, and serine, as well as essential AAs such as histidine, methionine, and threonine, were reduced considerably in both disease groups (Fig. 3A). In contrast, phenylalanine was only selectively decreased in NICM patients (Fig. 3A). We did not observe consistent changes in the levels of glutamate, glutamine, and branched-chain amino acids (BCAAs)—such as leucine/isoleucine, and valine within the nonfailing, ICM and NICM myocardia (Fig. 3A). However, we did observe a tendency toward a higher BCAA to total AA ratio, suggesting a relative accumulation of BCAA in both ICM and NICM hearts (Fig. 3B).

Our untargeted metabolomics detected several acetyl-CoA derivatives—acetylphosphate, *N*-acetylglutamine, and *N*-acetylglutamate—that did not show significant differences among the three groups. Interestingly, pyroglutamic acid (PGA) is a ubiquitous, yet understudied, natural AA derivative of glutamic acid or glutamine. Pyroglutamic acidosis has been associated with organ failure and mortality among patients with sepsis [16]. In end-stage failing ICM and NICM hearts, the levels of PGA were markedly lower compared to nonfailing hearts (Fig. 3A). Although lysine levels did not differ among the three groups, there were significant decreases in the levels of acetyllysine, an important metabolite involved in posttranslational acetylation of cardiac proteins and HF progression [17], in ICM and NICM hearts (Fig. 3A). Another metabolite in the lysine degradation pathway, 2-aminoadipate, was also significantly reduced in both HF groups (Fig. 3A). As previously reported [11,18], the levels of creatine, synthesized from glycine, arginine, and methionine, were significantly lower in ICM and NICM hearts compared to nonfailing hearts, coinciding with much reduced levels of glycine and methionine (Fig. 3A). We also observed reduced creatinine levels, a waste product of muscle metabolism, in ICM and NICM hearts (Fig. 3A). Histamine, an important signaling intermediate derived from histidine, was largely depleted. However, another histidine derivative, 1-methyl histidine, displayed a tendency for higher levels in both ICM and NICM hearts (Fig. 3A). The kynurenine pathway is the main route for tryptophan metabolism. Interestingly, the NICM myocardium showed reduced kynurenine levels compared with nonfailing hearts (Fig. 3A). Additionally, we observed that two urea cycle amino acid metabolites, ornithine and citrulline, were either trending higher or significantly elevated in both disease groups (Fig. 3A).

Disruption of the Homocysteine-Methionine (HM) cycle leads to elevated homocysteine (Hcy) levels, i.e., hyperhomocysteinemia (Fig. 3G). Several studies have shown a positive association between elevated total plasma Hcy and the risk of HF [8,19]. We, for the first time, identified that Hcy levels were increased in the ventricles of both ICM and NICM patients compared with nonfailing groups, with no significant differences between the two types of HF (Fig. 3C). This suggests that hyperhomocysteinemia is also present in end-stage failing hearts.

At last, we specifically examined the glutathione pool and its synthesis in failing hearts. Both the reduced (GSH) and oxidized glutathione (GSSG) levels in ICM and NICM hearts were significantly lower compared to the nonfailing hearts (Fig. 3D). Ultimately, there were no differences in the ratios of GSH/GSSG across groups (Fig. 3E). Glutathione is synthesized from cysteine and glutamate by the sequential action of glutamate-cysteine ligase (*GCL*), composed of the catalytic (*GCLC*) and modulatory (*GCLM*) subunits, and GSH synthetase (*GSS*) (Fig. 3G). Myocardial cysteine and glutamate levels were not significantly changed in ICM and NICM hearts (Fig. 3A). We only identified a slight upregulation of *GCLC* mRNA, but not *GCLM*, in NICM hearts. In contrast, no differences in the transulfuration pathway enzyme cystathionine gamma-lyase (*CTH*) and *GSS* expression were observed among the three groups (Fig. 3F). We did not analyze cystathionine β-synthase (*CBS*), the first and rate-limiting enzyme of the transsulfuration pathway that synthesizes cysteine, since it barely expresses in the heart (Fig. 3G). Overall, ICM and NICM hearts exhibit similar disruption of amino acid metabolism and glutathione balance.

### 3.4. Aberrant purine and pyrimidine metabolism in ICM and NICM hearts

**Fig. 4.**
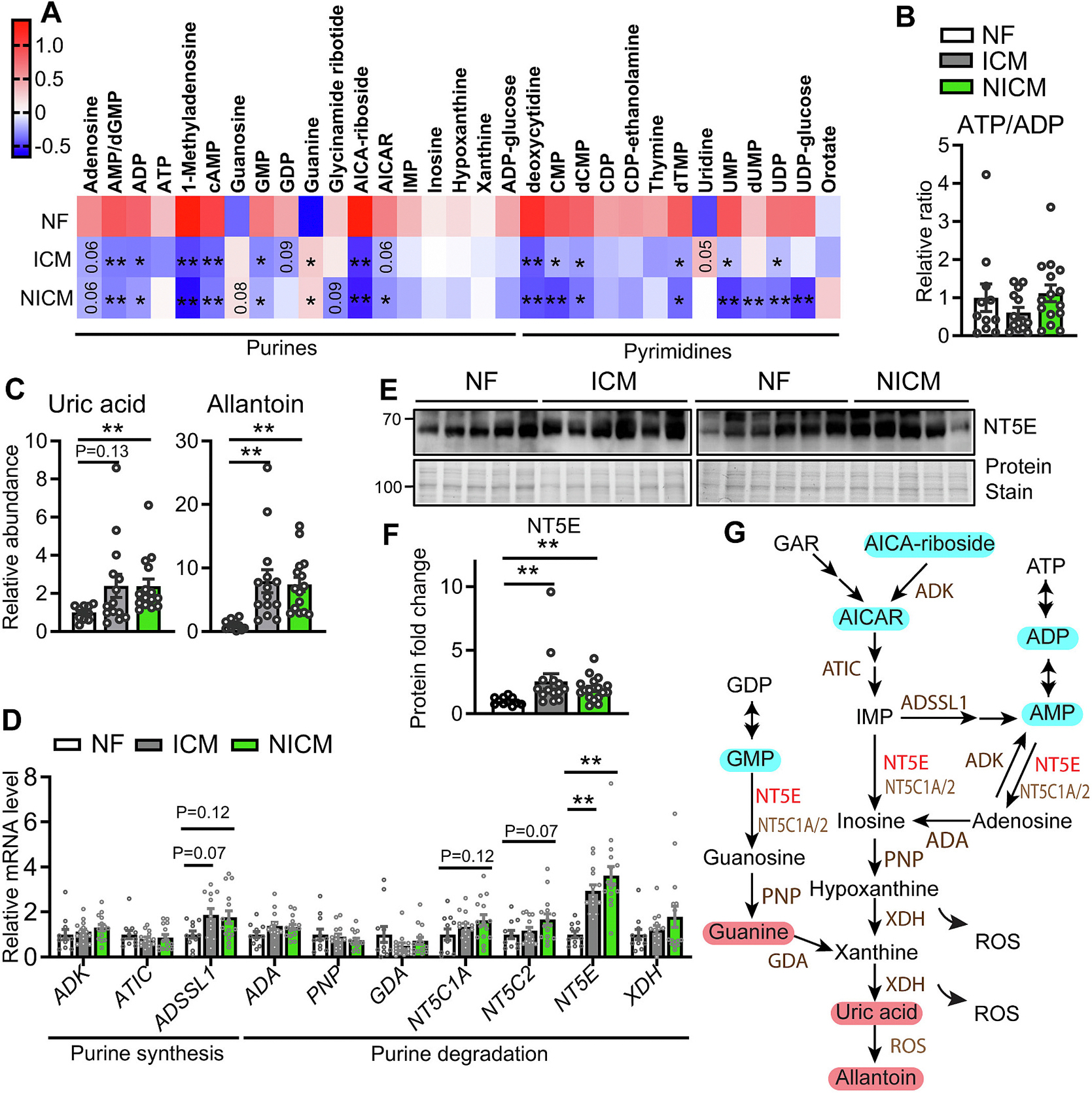
Deranged purine and pyrimidine metabolism in ICM and NICM hearts. A) Heatmap illustrating metabolites categorized into purines and pyrimidines and their derivatives. *: P < 0.05; **: P < 0.005. Unpaired t-tests. B) Relative ATP to ADP ratio. Kruskal-Wallis test with post hoc Dunn test for multiple comparisons. C) Relative abundances of purine degradation products (uric acid and allantoin) Kruskal-Wallis test with post hoc Dunn test for multiple comparisons. D) RT-PCR analysis of genes involved in purine synthesis and degradation. One-way ANOVA with Tukey’s post hoc test. *E*-F) Western blot and quantification of NT5E. Data were normalized to quantifications of stain-free bands. Kruskal-Wallis test with post hoc Dunn test for multiple comparisons. 11 nonfailing, 14 ICM, and 15 NICM hearts were analyzed. *: P < 0.05; **: P < 0.005. G) Schematic diagram of identified metabolites involved in purine synthesis and degradation pathways. Upregulated metabolites and genes are highlighted in red; downregulated metabolites are highlighted in blue.

Disruption of purine metabolism is also closely associated with heart failure [11,20]. In both ICM and NICM myocardia, adenosine levels tended to decrease, with significant reductions in AMP/dGMP and ADP (Fig. 4A). Surprisingly, ATP levels remained unchanged, as well as the ratios of ATP/ADP across the three groups (Figs. 4A–4B). Interestingly, we observed a decrease in GMP, while guanosine levels tended to rise, and guanine levels increased nearly 5-fold in both ICM and NICM hearts (Fig. 4A). These data suggest distinct metabolic pathways for adenylate and guanylate purines in end-stage failing hearts.

Notably, 1-methyladenosine, a purine derivative involved in m1A methylation of RNA that regulates various biological functions and pathogenesis [21], was significantly decreased in both diseased hearts compared to the nonfailing ones (Fig. 4A). Additionally, the levels of cAMP were reduced by approximately 65% in ICM and NICM myocardia relative to the nonfailing hearts (Fig. 4A), consistent with the finding of decreased cAMP synthesis in human failing hearts [22].

When examining the intermediate metabolites involved in de novo purine synthesis, we saw no noticeable changes in glycinamide ribotide (GAR) and inosine. However, there was a significant decrease in 5-aminoimidazole-4-carboxamide-1-β-d-ribofuranoside (AICAR) in NICM and a trend toward lower levels in ICM (Fig. 4A). Interestingly, AICA-riboside, a drug used to activate AMP-activated protein kinase (AMPK) after its phosphorylation by adenosine kinase (ADK) to AICAR, was also significantly reduced in failing hearts (Fig. 4A).

Purines are metabolized into hypoxanthine and xanthine by xanthine dehydrogenase/oxidase (gene name: *XDH*), leading to uric acid production and its further oxidation by free radicals to allantoin (Fig. 4G). Increased circulating levels of uric acid and allantoin have been positively linked to oxidative stress, the risk of HF, and poorer prognosis [23–25]. We observed that hypoxanthine and xanthine levels remained unchanged among the three groups. However, uric acid levels tended to be higher in ICM ventricles and were significantly increased in NICM hearts compared to nonfailing hearts (Fig. 4C). Importantly, allantoin levels were markedly upregulated in both ICM and NICM hearts compared to nonfailing hearts (Fig. 4C).

Next, we analyzed the expression of genes involved in purine synthesis and breakdown. The mRNA levels of 5-aminoimidazole-4-carboxamide ribonucleotide formyltransferase/inosine monophosphate cyclohydrolase (ATIC) and adenosine kinase (ADK) remained unchanged, but there was a trend toward higher expression of adenylosuccinate synthase 1 (ADSSL1), an essential enzyme for converting IMP to AMP, in ICM and NICM hearts compared to nonfailing controls (Fig. 4D). The mRNA levels of cytosolic 5′,3′-nucleotidase isoforms (NT5C1A and NT5C2), which regulate AMP, IMP, and GMP degradation, were not significantly different across groups. Interestingly, 5’-Nucleotidase (NT5E or CD73), a key extracellular enzyme that converts AMP and GMP into adenosine and guanosine (Fig. 4G), was significantly higher in ICM and NICM hearts at both mRNA (Fig. 4D) and protein levels (Figs. 4E–4F). Other major enzymes involved in purine breakdown, such as adenosine deaminase (ADA), purine nucleoside phosphorylase (PNP), and guanosine deaminase (GDA), showed no significant change (Fig. 4D). Although the expression and activity of xanthine dehydrogenase/oxidase have been reported to be elevated in failing hearts [26], we did not observe significant differences in *XDH* mRNA levels in either ICM or NICM myocardium (Fig. 4D).

Much less attention has been given to myocardial pyrimidine metabolism in end-stage failing hearts. We observed significant decreases in deoxycytidine, CMP, dCMP, and dTMP levels in both ICM and NICM hearts. In contrast, the levels of CDP, CDP-ethanolamine, thymine and orotate, a pyrimidine precursor, remained stable (Fig. 4A). Plasma uridine is linked to the risk of HF [27]. We noted a slight rise in uridine levels only in ICM ventricles (Fig. 4A). Importantly, UMP, dUMP, UDP, and UDP-glucose were significantly reduced in NICM hearts compared to nonfailing controls, with no notable differences between ICM and NICM hearts (Fig. 4A). Overall, our results reveal extensive disturbances in purine and pyrimidine metabolism in both ICM and NICM hearts.

### 3.5. Increased NAD+/NADH ratios and loss of energy transfer metabolites in ICM and NICM hearts

**Fig. 5.**
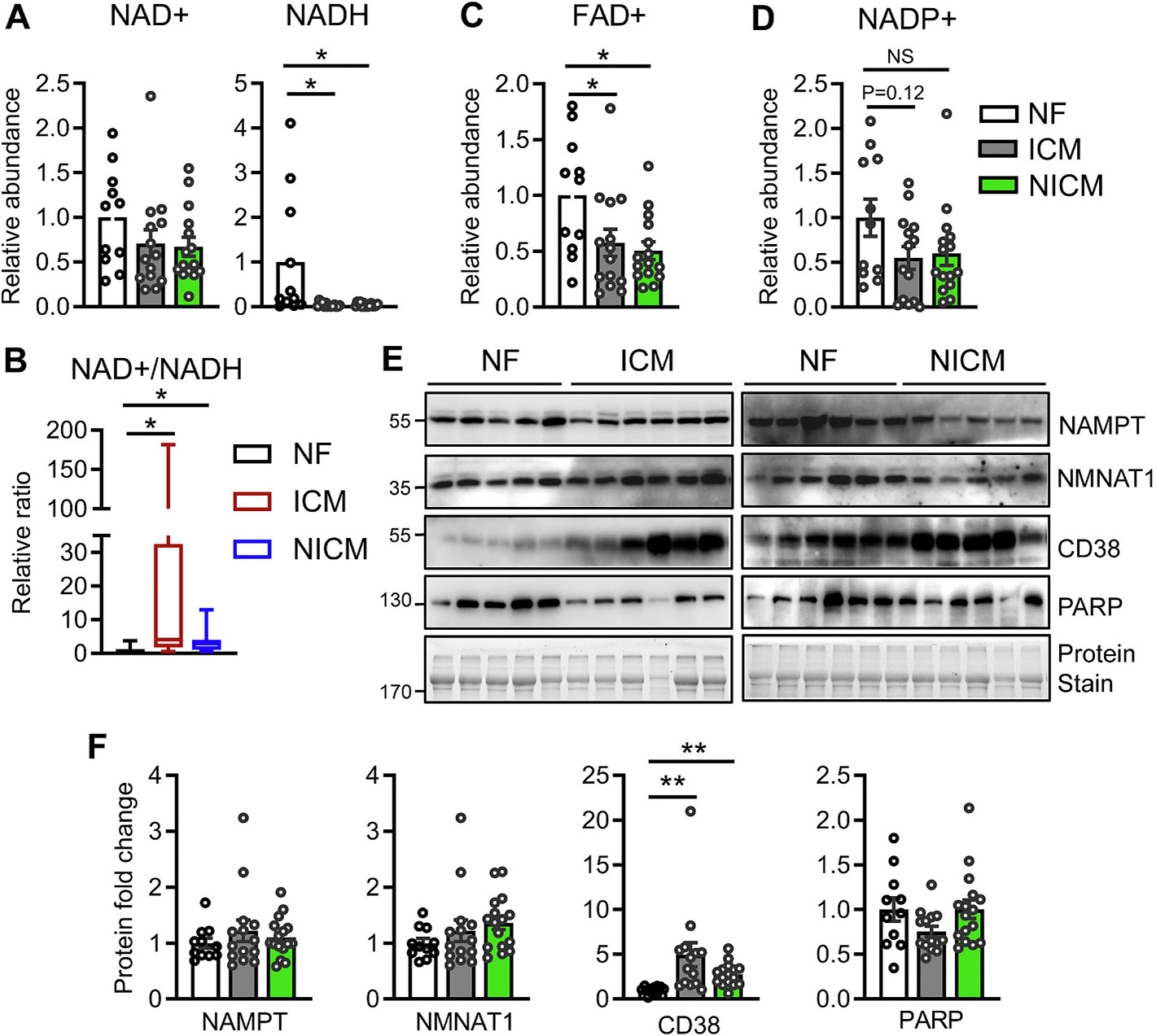
Elevated NAD+/NADH ratios and alterations in electron transfer metabolites in ICM and NICM hearts. A) Relative abundances of NAD+ and NADH. Kruskal-Wallis test with post hoc Dunn test for multiple comparisons. B) Whisk and box plot indicating the NAD+/NADH ratios based on the levels from untargeted metabolomic analyses, with whiskers indicating the minimum to maximum. Kruskal-Wallis test with post hoc Dunn test for multiple comparisons. C–D) Relative abundances of FAD+ (C) and NADP+ (D). One-way ANOVA with Tukey’s post hoc test. E-F) Western blot and quantification of NAD biosynthesis and consumption enzymes. Data were normalized to quantifications of stain-free bands. Kruskal-Wallis test with post hoc Dunn test for multiple comparisons. 11 nonfailing, 14 ICM, and 15 NICM hearts were analyzed. *: P < 0.05; **: P < 0.005.

Redox-active coenzymes like NAD, NADP, and FAD are crucial for cellular functions, including metabolism, energy generation, and antioxidant defense. Intracellular NAD exists in both oxidized (NAD+) and reduced (NADH) states. Our findings showed no differences in NAD+ levels across nonfailing, ICM, and NICM hearts (Fig. 5A). However, NADH levels were markedly lower in both ICM and NICM hearts (Fig. 5A). As a result, the NAD+/NADH ratio was unexpectedly higher in both ICM and NICM, with a more noticeable increase in ICM myocardium (Fig. 5B). Additionally, FAD+ levels decreased in both types of failing hearts (Fig. 5C). No significant changes were observed in NADP+ levels, and the reduced forms of FAD+ and NADP+ were not detected in our untargeted metabolomic analyses (Fig. 5D). When analyzing the protein expression of key enzymes involved in NAD+ synthesis and utilization, we found the levels of nicotinamide phosphoribosyltransferase (NAMPT) and nicotinamide nucleotide adenylyltransferase 1 (NMNAT1), which are crucial for NAD+ biosynthesis, remained unchanged across all three groups (Fig. 5E and F). In contrast, the protein expression of cyclic ADP-ribose synthase (CD38), a primary NADase degrading NAD+, was significantly upregulated in hearts with ICM and NICM (Fig. 5E and F). The levels of poly(ADP-ribose) polymerase (PARP), another NAD + -consuming enzyme, did not differ among the groups. These findings indicate that despite higher NAD+ consumption by CD38, ICM and NICM hearts maintain NAD+ levels but exhibit reduced levels of other energy-transfer metabolites.

### 3.6. Altered oxidative defense enzymes in ICM and NICM hearts

**Fig. 6.**
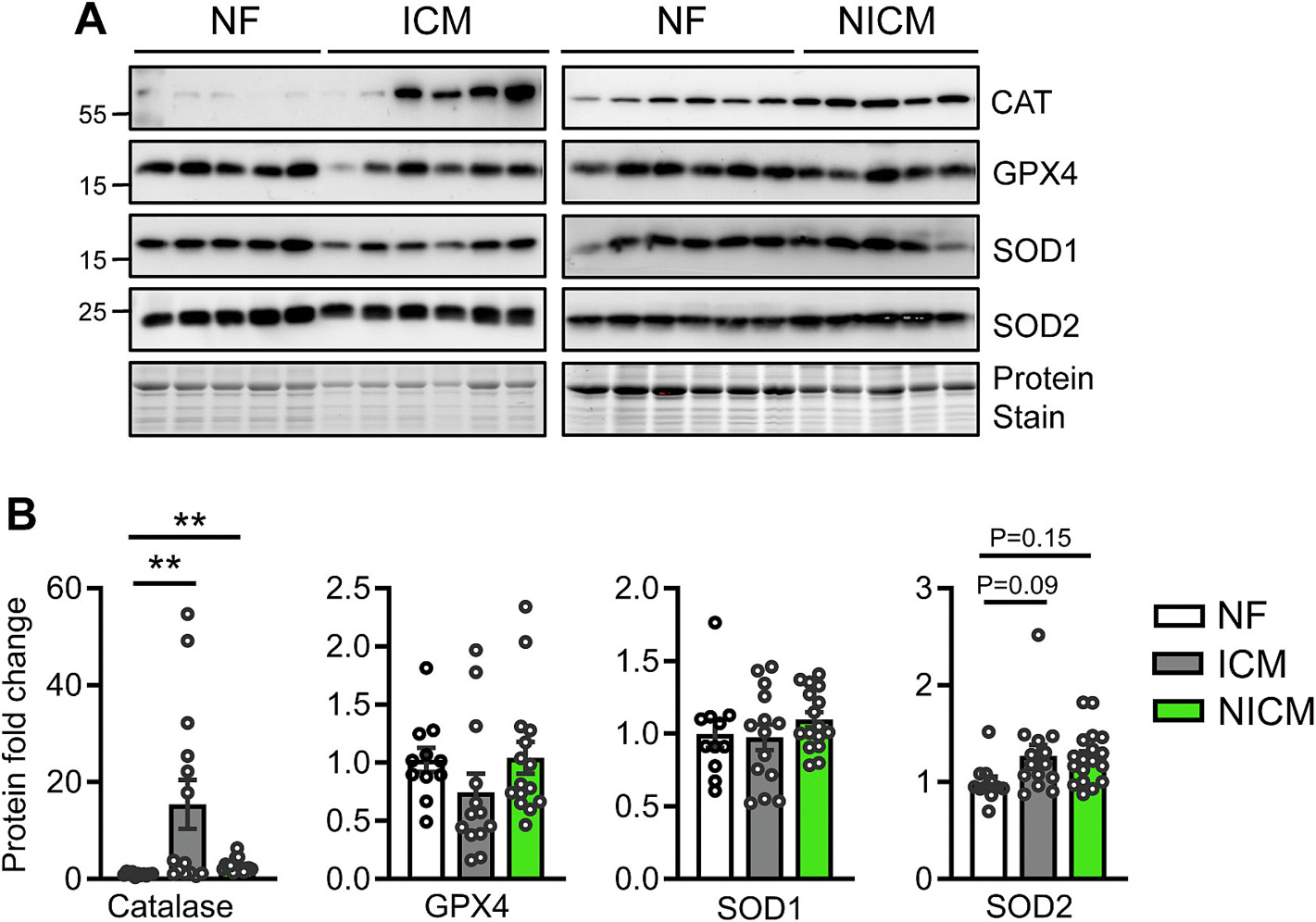
Alterations in the expression of antioxidant enzymes in ICM and NICM hearts. A-B) Western blot and quantification of antioxidant enzymes. Data were normalized to quantifications of stain-free bands. Kruskal-Wallis test with post hoc Dunn test for multiple comparisons. 11 nonfailing, 14 ICM, and 15 NICM hearts were analyzed. *: P < 0.05; **: P < 0.005.

Both ICM and NICM hearts exhibit high homocysteine, reduced glutathione, elevated uric acid and allantoin, and a higher NAD+/NADH ratio, all of which are strong indicators of oxidative stress. We next examined the expression of enzymes involved in the myocardial antioxidant defense system, including glutathione peroxidase (GPx), superoxide dismutase, and catalase. We found that catalase protein expression was significantly upregulated in both ICM and NICM hearts (Figs. 6A–6B). There were no changes in GPX4 or SOD1 protein expression, whereas a trend toward higher SOD2 expression was observed in ICM and NICM hearts (Figs. 6A–6B). These data indicate altered expression of antioxidant enzymes in end-stage failing hearts.

## 4. Discussion

Our metabolomic analysis of end-stage ischemic and nonischemic HFrEF confirmed many previously identified metabolomic alterations, including changes in glucose, amino acids, purines, oxidative stress, and antioxidant enzymes. Additionally, we uncovered several novel findings. First, myocardial metabolomic profiles did not differ between end-stage ICM and NICM, despite notable differences in diabetes and hypertension rates. Second, rather than upregulation, we observed downregulation of myocardial *O*-GlcNAcylation in failing hearts. Third, NAD+ levels were not depleted; instead, NADH was reduced, leading to a higher NAD+/NADH ratio in end-stage heart failure. Overall, our study provides new insights into myocardial metabolic reprogramming in human end-stage failing hearts.

### 4.1. Cardiac energetics

The failing heart is often described as “energy-starved,” reflecting impaired energy production and usage. In advanced HF, glucose metabolism may also be limited [28,29]. Our analysis detected only a few glycolysis intermediates, offering limited insights into glucose metabolism in end-stage HFrEF. Notably, glucose-6-phosphate and lactate levels remained stable between failing and nonfailing hearts, despite higher circulating pyruvate and lactate levels linked to HF and serving as prognostic markers of mortality [30,31]. Unlike prior studies on end-stage HFrEF [11] or HFpEF [12], TCA cycle metabolites in our cohort did not show a significant decrease. The maintained TCA cycle metabolites may be replenished by breaking down other substrates, such as amino acids, as indicated by decreased amino acid levels in our HFrEF group. We confirmed a specific reduction in adenylate purines, crucial for ATP maintenance. However, unlike earlier research [11], ATP levels and ATP/ADP ratios did not differ significantly between nonfailing and diseased hearts [11]. The lack of phosphocreatine (pCr) data prevents direct comparisons of the pCr/ATP ratio; however, lower creatine levels still suggest compromised creatine kinase shuttle and energy reserves in our HFrEF group. These findings indicate an energy crisis rather than an energy-starved state in our HFrEF patients, regardless of etiology.

Another evidence supporting the energy crisis in our HFrEF group is the significant decrease in NADH and FAD+ levels (Figs. 5A–C). Mitochondrial oxidative phosphorylation relies on electron carriers like NADH and FADH2 for ATP production. A decline in NADH and FAD+ suggests less fuel for ATP synthesis, indicating impaired mitochondrial function. Interestingly, previous study shows that NAD+ levels and the NAD+/NADH ratio often decrease in heart failure [32]. This “NAD+ redox imbalance” is believed to result from excessive NAD+ consumption by enzymes such as PARP and CD38 during cardiac stress [33]. We identified elevated CD38 levels in both ICM and NICM hearts, which are known to be associated with decreased energy availability and function [34]. Nicotinic acid riboside (NAR) supplementation has been clinically tested to improve heart failure symptoms [35]. However, our findings challenge the notion that NAD+ levels are reduced in end-stage HF. We mainly observed a decrease in NADH with maintained NAD+ in end-stage HFrEF, leading to a higher NAD+/NADH ratio (Figs. 5A–B). Notably, levels of both NAD+ and NADH remained unchanged in the myocardium of another HFrEF cohort [11]. This variability might be due to cohort-specific factors or to differences in sample size. Our study indicates that the net NADH level could be a more reliable marker of mitochondrial deficiency in HF.

### 4.2. Ancillary glucose metabolism pathway

*O*-GlcNAcylation and its regulatory enzymes are elevated in patients with aortic stenosis and in various animal models of hypertension, hypertrophy, and HF [36,37]. Sustained activation of the HBP and *O*-GlcNAcylation is associated with cardiac dysfunction, as demonstrated in genetic mouse models with abnormal *O*-GlcNAcylation levels [38]. However, we identified decreased UDP-GlcNAc levels and reduced O-GlcNAcylation in our end-stage HFrEF cohorts (Fig. 2). While reduced UDP-GlcNAc levels align with previous research [11,39], reduced cardiac *O*-GlcNAcylation may suggest that it is more sensitive to nutrient and metabolic fluxes, particularly UDP-GlcNAc levels. Elevated expression of *O*-GlcNAcylation enzymes might serve as a compensatory response to maintain specific O-GlcNAc levels and support cellular homeostasis. Nonetheless, further studies are needed to better understand the exact role of *O*-GlcNAcylation in human hearts.

Among metabolites in the PPP pathway, only diet-derived D-gluconate levels increased in ICM and NICM myocardium, consistent with earlier research [11]. G6PD1 and NADPH oxidase activities are elevated in failing human hearts [40]. However, our findings showed decreased *G6PD1* mRNA levels in failing hearts. Interestingly, *G6pd* knockout has been shown to worsen cardiac function after ischemia–reperfusion injury in mice [41]. Currently, it is unclear whether G6PD1 downregulation is an adaptive or maladaptive response and whether its reduction contributes to heart failure.

### 4.3. Amino acid metabolism

Our study showed reductions in nonessential and essential AAs in myocardial tissue from end-stage HFrEF, consistent with previous reports [11,12]. However, unlike these studies, myocardial BCAA levels did not increase but remained unchanged in our HFrEF group. We observed a higher myocardial BCAA/AAs ratio, consistent with the increased plasma BCAA/AA ratio in HF [42]. Additionally, histidine was the most significantly downregulated AA, possibly due to its antioxidant role, and chronic inflammation in HF further reduced its levels. Interestingly, two major histidine derivatives changed in opposite directions: 1-methyl-histidine increased while histamine decreased. This indicates a more complex regulation of histidine metabolism in HF. Plasma 1-methyl-histidine levels are positively associated with acute decompensated and chronic HF [9]. The specific roles of 1-methylhistidine and its synthesizing enzyme, METTL9, in cardiac function are currently being intensively studied [43].

Another interesting finding is hyperhomocysteinemia in the myocardium itself in HF patients. This increase may mirror changes in the plasma of HF patients [8,19,44], which have typically been linked to liver CBS dysfunction or issues with vitamin B12 metabolism in the liver [45]. Nonetheless, hyperhomocysteinemia could contribute to the progression of HF through collagen buildup, increased myocardial stiffness, and reduced contractile function, etc. [46].

Myocardial metabolomics show limited overlap with plasma metabolomics in both HFrEF and HFpEF [12]. Our research also identified inconsistencies between these profiles. Elevated plasma kynurenine levels are associated with increased mortality in HF patients [47]. Yet kynurenine pathway metabolites decreased in HFrEF. Similarly, lower plasma urea cycle metabolites have been linked to coronary artery disease, atrial fibrillation, or HF risk [48,49]. However, urea cycle metabolites were consistently elevated in NICM HFrEF (Fig. 3 and [11]). Such accumulation in the myocardium could be due to impaired urea cycle activity, as observed in patients with compromised cardiac function [29].

### 4.4. Purine and pyrimidine metabolism

We also found that intermediates in purine synthesis were depleted, while degradation products increased in both ICM and NICM HFrEF compared to nonfailing myocardium, consistent with previous reports [11,50]. Uric acid and other adenine nucleotide degradation products are elevated in the plasma of patients with various heart diseases [20]. Although the mRNA levels of key genes involved in de novo purine synthesis did not change, the decreases in AICA Riboside and AICAR suggest possible allosteric inhibition. Moreover, lower AICAR levels may support therapies that activate AMPK with AICAR, potentially improving heart function, as AICAR treatment has shown benefits in heart failure models [51].

Our findings support that increased purine degradation may mainly occur through allosteric effects rather than changes in gene expression, since the two cytosolic 5′3’-nucleotidases (NT5C1A and NT5C2) were not transcriptionally regulated [11]. However, we specifically identified elevated ecto-5′-nucleotidase (NT5E) enzyme, which is associated with higher blood adenosine levels and could indicate the presence or severity of chronic HF [52]. The effect of extracellular purine breakdown on intracellular purine depletion in end-stage HFrEF remains unclear. Interestingly, we consistently identified upregulated guanine in the myocardium of HF patients (Fig. 4C and [11,50]). While no changes in guanine metabolic genes were observed, the mechanisms and consequences of this upregulation need further investigation.

Another distinct finding is a broad depletion of pyrimidine and its derivatives in end-stage HFrEF (Fig. 4A), which was not previously reported [11]. Meanwhile, bioactive metabolites, such as cAMP, 1-methyladenosine, and deoxycytidine, were markedly downregulated. These metabolites play crucial roles in signaling reprogramming and epigenetic changes within the myocardium affected by HFrEF. Reduced cAMP levels in human failing hearts have been documented [11], supporting the notion of impaired cAMP signaling in advanced HFrEF. Synthetic deoxycytidine analogs are utilized in preclinical models to enhance DNA methylation, which is linked to the development and potential reversal of heart failure phenotypes [53]. The function of RNA m1A methylation in cardiac disease remains largely unexplored, underscoring the need for further research to better understand its involvement in HF pathogenesis.

### 4.5. Cardiac oxidative stress and antioxidant system

Human failing myocardium exhibits increased oxidative stress [54], yet the core antioxidant and detoxification mechanisms of the heart remain poorly understood. Our research identified high homocysteine levels, a significant reduction in total glutathione, elevated uric acid and allantoin, and a higher NAD+/NADH ratio, all of which are strong indicators of oxidative stress. The lowered GSH and GSSG in our HFrEF group may reflect depletion due to oxidative stress, while the increased *GCLC* mRNA in NICM might be a compensatory mechanism. During heart failure, xanthine oxidase levels and activity rise with purine breakdown, potentially establishing a “vicious cycle” of oxidative damage [26]. Although we did not observe increased *XDH* mRNA, XDH/XO activity could be elevated in end-stage HFrEF, warranting further study. Changes in key antioxidant enzymes have shown inconsistent patterns in expression and activity [54,55]. We detected a specific increase in catalase protein levels, whereas SOD and GPX4 remained unchanged (Figs. 6A–B). These findings suggest a compensatory response to oxidative stress in human end-stage heart failure.

In summary, our data show no distinct differences in metabolomic profiles between end-stage ICM and NICM. However, it confirms many significant disruptions in amino acids and purines, along with increased oxidative stress and higher Catalase expression, in end-stage HFrEF. We also identify reduced *O*-GlcNAcylation, decreased pyrimidines, a higher NAD+/NADH ratio, and various shifts in bioactive metabolites in end-stage HF. These insights deepen our understanding of metabolic disruptions in the human failing heart.

### 4.6. Limitations

It is well known that metabolomic profiling provides only a static snapshot of metabolite levels and does not capture metabolic flux, which requires studies with isotope tracers. Although our samples include both ICM and NICM cardiomyopathy, each group has a small sample size, and only male samples were used. This may limit our ability to detect more consistent changes and to compare sex differences in cardiac metabolites. Additionally, our results were limited by using an in-house metabolite library of ~300 standards. This increased confidence in metabolite reporting, as these metabolites are identified rather than annotated. However, future work would benefit from a fully untargeted metabolomics approach using MS2 fragmentation data for metabolite annotations. Furthermore, we did not perform lipidomics to identify lipids and ketone bodies, which restricts our capacity to evaluate fatty acid and ketone body utilization in this cohort. Lastly, all studies were conducted in end-stage HF, restricting our interpretation of these metabolic changes to the context of HF development.

## Supplementary Material

1

## Figures and Tables

**Table 1:** Clinical characteristics for nonfailing controls (*n* = 11) and patients with ischemic cardiomyopathy (*n* = 14) and nonischemic cardiomyopathy (*n* = 15). Endomyocardial tissues were obtained from Duke Human Heart Repository (DHHR).

	Nonfailing (n = 11)	ICM (n = 14)	NICM (n = 15)

Sex	male	male	male
Age, y	45.5 ± 18.8	62.8 ± 6.8*	53.1 ± 11.7
Race, White, n (%)	8 (72.7)	9 (64.3)	6 (40)
Race, Black, n (%)	3 (27.3)	5 (35.7)	9 (60)
Body mass index (kg/m2)	26.3 ± 4.8	28.2 ± 6.3	30.5 ± 6.6
**Comorbidities, n (%)**			
History of smoking	8 (72.7)	11 (78.6)	10 (66.7)
Type 2 diabetes mellitus	0 (0)	11 (78.6)	3 (20)
Hyperlipidemia	0 (0)	10 (71.4)	2 (13.3)
Chronic kidney disease	0 (0)	8 (57.1)	4 (26.7)
Hypertension	5 (45.5)	12 (85.7)	*4 (26.7)*
Atrial fibrillation	0 (0)	4 (28.6)	5 (33.3)
**Drugs, n (%)**			
Insulin	0 (0)	6 (42.8)	3 (20)
Diuretic	0 (0)	9 (64.2)	10 (66.7)
β-Blocker	0 (0)	5 (35.7)	10 (66.7)
Mineralocorticoid antagonist	0 (0)	7 (50)	7 (46.7)
Hydralazine	0 (0)	0 (0)	3 (20)
Angiotensin-converting enzyme inhibitors/angiotensin receptor blocker /angiotensin receptor neprilysin inhibitor	0 (0)	3 (21.4)	3 (21.4)

* *P* < 0.05 versus nonfailing control. Kruskal-Wallis test with post hoc Dunn test for multiple comparisons.

## Appendix A. Supplementary data

Supplementary data to this article can be found online at https://doi.org/10.1016/j.yjmcc.2026.02.008.
