# Supplementary material for: Myocardial metabolic remodeling in human end-stage ischemic and non-ischemic cardiomyopathy
Source: J Mol Cell Cardiol. Author manuscript; Available in PMC 2026 Jul 9. (PMC13347790; doi:10.1016/j.yjmcc.2026.02.008)
Supplement: 1 [file NIHMS2179291-supplement-1.pdf]

Supplemental Materials

Myocardial Metabolic Remodeling in Human End-Stage Ischemic and Non-Ischemic Cardiomyopathy

Hongyi Zhou[1], Courtney Jayde Christopher[2], Katarina Jones[3], Michelle Mendiola Pla[4]; Ryan T. Gross[4], Gabriel Esmailian[4], Shawn Robert Campagna[2], Dawn E. Bowles[4], Weiqin Chen[1*]

Supplemental Materials

Untargeted quantitative metabolomics:

Tissues were kept at 4°C, pre-weighed, and homogenized. The exact mass of tissue used for each sample was recorded and used for normalization. Roughly 25 mg of tissue was aliquoted for each sample, and water-soluble metabolites were extracted using an acidic acetonitrile extraction procedure adapted from Rabinowitz and Kimball[1] using 1.5 mL of 4:4:2 acetonitrile: methanol: water with 0.1 M formic acid.[2] All solvents were HPLC grade. The supernatant collected from each sample was dried under nitrogen, then 300 μL of LC-MS grade water was added to each sample before mass spectral analysis.

A previously described ultra-high-performance liquid chromatography high-resolution mass spectrometry (UHPLC-HRMS) method was used for untargeted metabolomics analysis[3] using an UltiMate 3000 RS autosampler (Dionex, Sunnyvale, CA, USA), Synergi 2.6 μm Hydro RP column (100 mm × 2.1 mm, 100 Å; Phenomenex, Torrance, CA, USA), an UltiMate 3000 pump (Dionex), and Exactive Plus Orbitrap mass spectrometer (Thermo Fisher Scientific, Waltham, MA, United States). A previously described 25-minute gradient elution, reverse-phase ion-pairing method with a water:methanol solvent system and a tributylamine ion-pairing reagent was used for chromatographic separation.[4] Metabolites were ionized via negative mode electrospray ionization (ESI) prior to full scan mass spectral analysis as previously described.[3]

Lipid samples were also extracted and analyzed at the BMSC at the University of Tennessee Knoxville. Homogenized and pre-weighed tissue samples were extracted at room temperature using a modified Bligh and Dyer protocol.[5] Prior to adding extraction solvent, each sample was spiked with 10 μL of SPLASH LIPIDOMIX (Avanti Research, Alabaster, AL, USA) isotopically labeled internal standards. The internal standards and the exact mass of tissue used for extraction were used to normalize the data. After the addition of standards, 750 μL of a 1:2 chloroform:methanol mixture, 250 μL of chloroform, and 250 μL of water were added to each

sample. The samples were thoroughly mixed before centrifugation at 4500 x g for 10 minutes. The lower organic layer was then collected, and the remaining aqueous layer was re-extracted with 250 µL chloroform. The combined organic layers were then combined and dried under a steady stream of N_2 until resuspension and analysis. Immediately before mass spectral analysis, the dried extracts were redissolved in 300 µL of 9:1 methanol: chloroform and then diluted 10-fold into acetonitrile. All solvents used were LC-MS grade, except chloroform, which was ACS grade.

Metabolomics data processing

Raw mass spectral files were converted to mzML files using a package from ProteoWizard, msConverter.[6] All mzML files were imported into an open-source software, metabolomics analysis and visualization engine (El-MAVEN), where metabolites were manually identified using an in-house library of ~300 metabolites based on exact mass (±5 ppm) and retention time (±2 min).[2, 7, 8] Lipids were manually identified by exact mass (±5 ppm) and retention time (±1 min) based on representative lipids from each lipid headgroup and class. Both metabolite and lipid peaks were integrated, and raw peak intensities for identified metabolites and lipids were exported from El-MAVEN to a CSV file. Prior to statistical analysis, raw spectral data were normalized by the mass of the sample used during extraction.

Metabolomics statistical analysis

The normalized data were imported into MetaboAnalyst 5.0, filtered using the interquartile range (IQR), log-transformed, and Pareto-scaled.[9] Partial least squares discriminant analysis (PLS-DA) was performed in MetaboAnalyst 5.0, where variable importance in projection (VIP) scores were assigned to each metabolite or lipid to indicate the contribution of each analyte to the separation between experimental groups. VIP scores > 1 indicate that a metabolite significantly contributes to the separation of groups in the PLS-DA model. Heatmaps were constructed using

R (version 1.0.153), which display $\log_2$ fold changes and p-values calculated based on Student's t-test.

Western blotting

Human heart tissues were homogenized in a lysis buffer comprising 25 mM Tris-HCl (pH 7.4), 150 mM NaCl, 2 mM EDTA, 1% Triton X-100, and 10% glycerol, with the freshly added protease inhibitor cocktail (Sigma) and phosphatase inhibitor cocktails (Fisher Scientific). Protein concentrations were determined via the Bradford protein assay (Bio-Rad). Equivalent amounts of protein homogenates were separated by SDS-PAGE and processed for standard Western immunoblotting. The membranes were incubated with specific antibodies, developed using the ECL chemiluminescence system, and imaged using an AMERSHAM Imager 600 (Cytivia). Densitometry analysis was performed using ImageQuant TL (Cytivia), and stain-free images were quantified in ImageJ. The following antibodies were used: CD38 (Proteintech, 60006-1-Ig), GFPT1 (Proteintech, 14132-1-AP), NAMPT (Proteintech, 11776-1-AP), NMNAT1 (Proteintech, 11399-1-AP), NT5E/CD73 (Proteintech, 12231-1-AP), OGT (Proteintech, 11576-2-AP), O-linked-N-Acetyl-glucosamine (Abcam, ab2739); PARP (Cell Signaling Technology, 9542), SOD1 (Proteintech, 10269-1-AP); SOD2 (Proteintech, 24127-1-AP), GPX4 (Abcam, ab125066), Catalase (Proteintech, 21260-1-AP).

Supplemental Table 1. List of Primers for human genes analyzed by RT-PCR analysis

Gene name	5' forward primer sequence	3' reverse primer sequence
ADA	TCCAAAGTGGAGCCAATCCC	TACACAGCTCCACCACCTTG
ADK	TCGAATATCATGCTGGTGGCT	GCACAAGTTCCTGTTGGCTG
ADSSL1	ATCATCAACACCAAGGCCGT	AGCCTCTTCTCCCAGTCCTT
ATIC	AACTGTTGAGGAGGCTGTGG	TGGCGTCTAGTCTCCAAGGA
CTH	TGAAGCGTCAGTGTACAGGT	AGGCTTTCGAATCCTCCCAA
G6PD1	CTACCGCATCGACCACTACC	TGTTGTCCCGGTTCCAGATG
GCLC	TGTGGTGTTTGTGGTACTGC	AACCATCCACCACTGCATTG
GCLM	TCCTGCTGTGTGATGCCA	GTGCGCTTGAATGTCAGGAA
GDA	CCTGCAGAACACAGATTCCA	GGCAAGGAGCAGAGATGAGT
GFPT1	GATGCCGGCGTTTGATTCTT	TCCACCATCACAGGCAACTC
GSS	CTGGCTGAGGGAGTATTGCT	ACAGCATCCACTAGCAGGTT
NT5C1A	CAGCTGGAACATGAGAACGA	CATGAGGACGATGTCGAAGA
NT5C2	GGAGTTGCTCAGCTTTGCTT	AAATCCATGTGCACAGACCA
NT5E	CCATGGAAATCCCATTCTTC	GATTGAGAGGAGCCATCCAG
OGT	TGCAACCTAGCCAATGCTCT	ACAAGCGAACTGCCTCTTCA
PNP	ACTGTGGCAGAATGTCGTGT	TTCTTCATGGTTGGCCTTCT
RPLP0	GCAATGTTGCCAGTGTCTGT	AGATGGATCAGCCAAGAAGG
XDH	CACTCAGAGGCAGCTTTCCA	TTCTCTTGGCCCAGCTTCTG

References

[1] J.D. Rabinowitz, E. Kimball, Acidic acetonitrile for cellular metabolome extraction from Escherichia coli, Anal Chem 79(16) (2007) 6167–73.

[2] L.O. Byerley, K.M. Gallivan, C.J. Christopher, C.M. Taylor, M. Luo, S.E. Dowd, et al., Gut Microbiome and Metabolome Variations in Self-Identified Muscle Builders Who Report Using Protein Supplements, Nutrients 14(3) (2022).

[3] W. Lu, M.F. Clasquin, E. Melamud, D. Amador-Noguez, A.A. Caudy, J.D. Rabinowitz, Metabolomic analysis via reversed-phase ion-pairing liquid chromatography coupled to a stand alone orbitrap mass spectrometer, Anal Chem 82(8) (2010) 3212–21.

[4] J.V. Bazurto, S.P. Dearth, E.D. Tague, S.R. Campagna, D.M. Downs, Untargeted metabolomics confirms and extends the understanding of the impact of aminoimidazole carboxamide ribotide (AICAR) in the metabolic network of Salmonella enterica, Microb Cell 5(2) (2017) 74–87.

[5] E.G. Bligh, W.J. Dyer, A rapid method of total lipid extraction and purification, Can J Biochem Physiol 37(8) (1959) 911–7.

[6] L. Martens, M. Chambers, M. Sturm, D. Kessner, F. Levander, J. Shofstahl, et al., mzML--a community standard for mass spectrometry data, Mol Cell Proteomics 10(1) (2011) R110 000133.

[7] M.F. Clasquin, E. Melamud, J.D. Rabinowitz, LC-MS data processing with MAVEN: a metabolomic analysis and visualization engine, Curr Protoc Bioinformatics Chapter 14 (2012) Unit14 11.

[8] E. Melamud, L. Vastag, J.D. Rabinowitz, Metabolomic analysis and visualization engine for LC-MS data, Anal Chem 82(23) (2010) 9818–26.

[9] Z. Pang, G. Zhou, J. Ewald, L. Chang, O. Hacariz, N. Basu, et al., Using MetaboAnalyst 5.0 for LC-HRMS spectra processing, multi-omics integration and covariate adjustment of global metabolomics data, Nat Protoc 17(8) (2022) 1735–1761.

Figure 2C

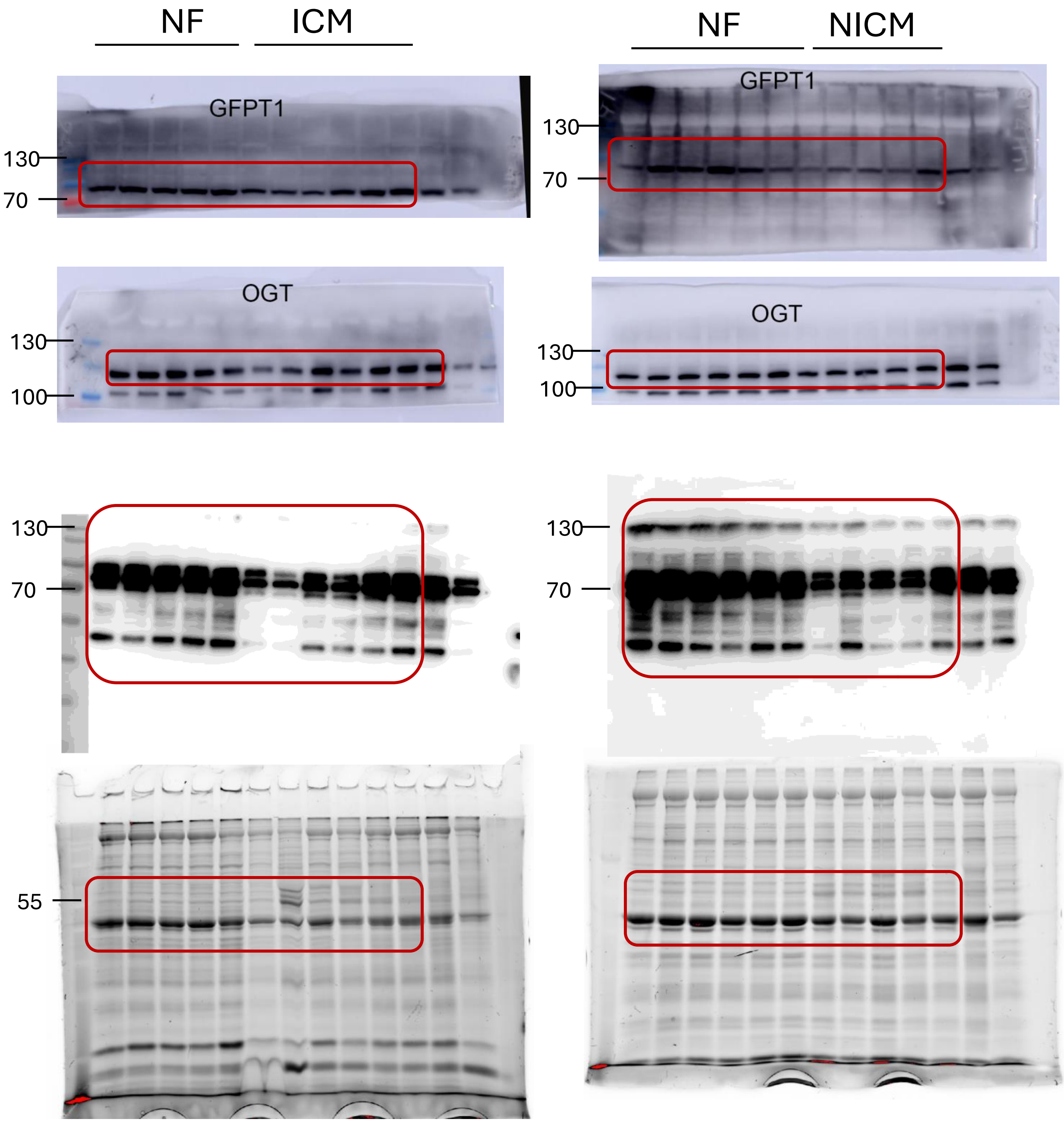
NF
ICM
NF
NICM
GFPT1
130
70
GFPT1
130
70
OGT
130
100
OGT
130
100
130
70
130
70
55

Figure 4E

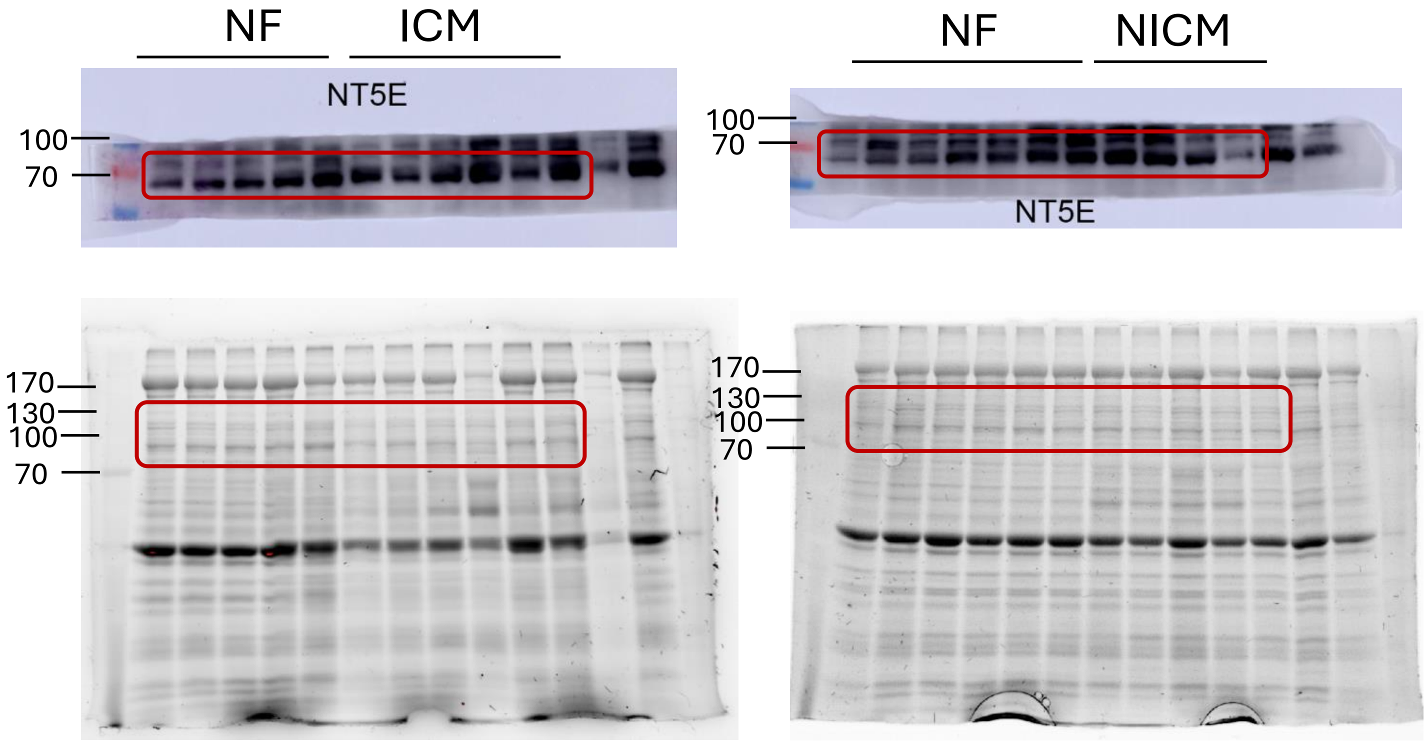
NF
ICM
NT5E
100
70
NF
NICM
100
70
NT5E
170
130
100
70
170
130
100
70

Figure 5E

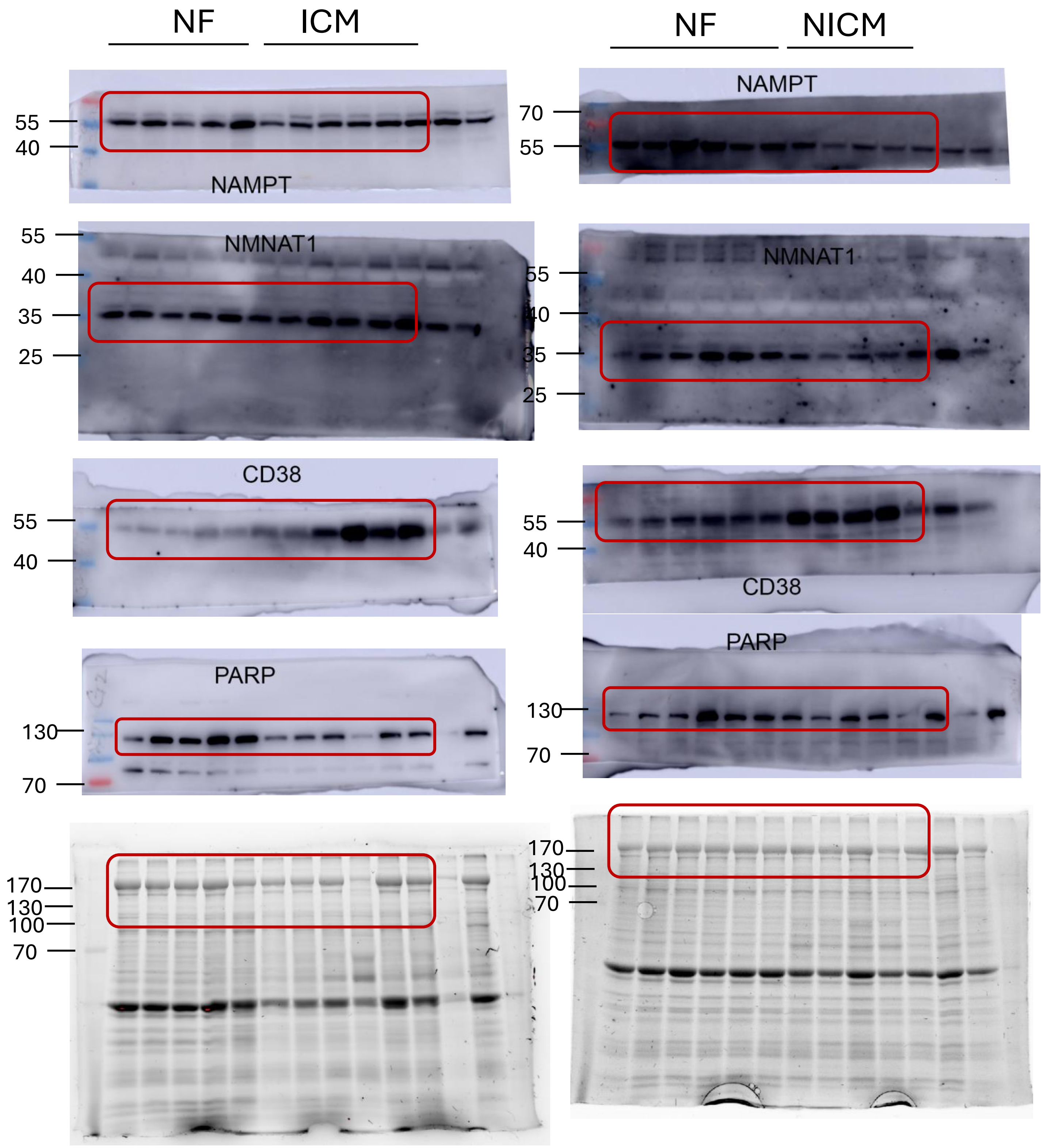
NF
ICM
NF
NICM
NAMPT
55
40
NAMPT
70
55
NMNAT1
55
40
35
25
NMNAT1
55
40
35
25
CD38
55
40
55
40
CD38
PARP
130
70
PARP
130
70
170
130
100
70
170
130
100
70

Figure 6A

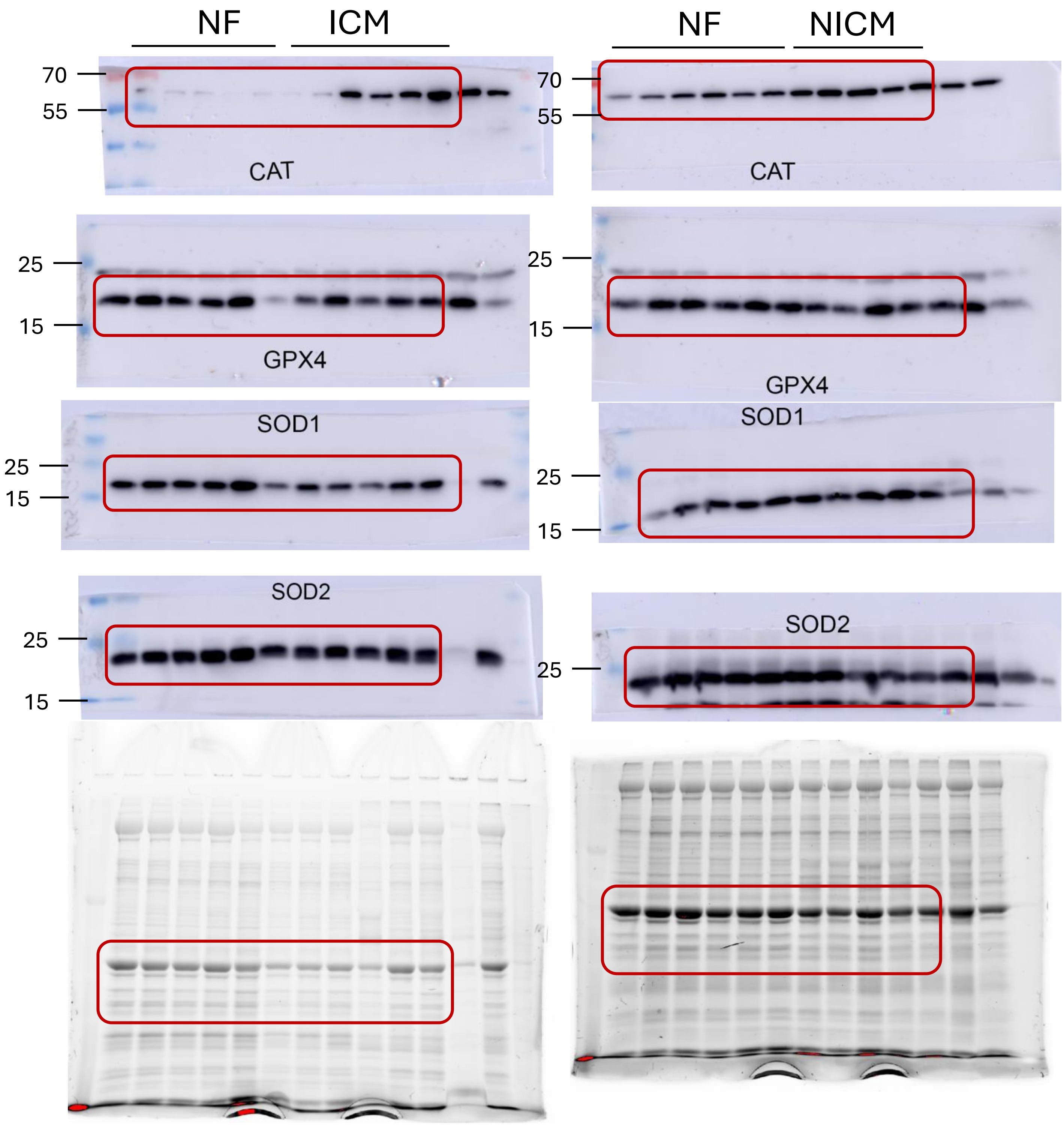
NF
ICM
NF
NICM
70
55
CAT
70
55
CAT
25
15
GPX4
25
15
GPX4
SOD1
25
15
SOD1
25
15
SOD2
25
15
SOD2
25
